# Unveiling the global impact of hypertensive heart disease among individuals aged ≥ 65 years: metabolic risk factors and future projections for 2050

**DOI:** 10.3389/fnut.2025.1611078

**Published:** 2025-11-06

**Authors:** Ning An, Dingding Qian, Weifeng Xu, Bingyu Wang

**Affiliations:** Department of Cardiology Vascular Internal Medicine, Ningbo Medical Center Lihuili Hospital, Ningbo University, Ningbo, China

**Keywords:** hypertensive heart disease, Bayesian age-time-cohort prediction model, public health nutrition, frontier analysis, metabolic risks

## Abstract

**Background:**

Hypertensive heart disease (HHD) poses a substantial global public health burden, particularly among older adults. Characterizing its epidemiological profile and projecting future trends are crucial for informing targeted prevention and management strategies.

**Methods:**

Utilizing global burden of disease (GBD) 2021 data spanning 1990–2021, we estimated age-standardized rates of prevalence, mortality, and disability-adjusted life-years (DALYs) for HHD in individuals aged ≥65 years across 204 countries and territories grouped into 21 region. We employed multiple analytical approaches: Joinpoint regression analysis to evaluate changes in age-standardized rates over time. Assessment of the proportion of HHD mortality and DALYs attributable to specific risk factors, quantified using population attributable fractions (PAFs) based on GBD risk-outcome associations and theoretical minimum risk exposure levels (TMRELs). Identification of key drivers (population growth, aging, epidemiological changes) underlying observed changes in HHD deaths and DALYs globally. Application of the Bayesian age-period-cohort (BAPC) model to evaluate age, period, and cohort effects on HHD risk and to project future incidence and mortality up to 2050. Quantification of socioeconomic disparities in HHD burden using the slope index of inequality (SII) and concentration index (CI), evaluated in relation to the socio-demographic index (SDI). Frontier analysis assessed the association between HHD burden and SDI levels. Identification of regions with the largest potential for reducing HHD burden relative to their SDI peers. Estimates are presented as means with 95% uncertainty intervals (UIs).

**Results:**

In 2021, the global age-standardized prevalence rate of HHD was 148.32 (95% UI: 117.32, 186.28) per 100,000 population. The age-standardized mortality rate was 16.32 (95% UI: 13.76, 18.01) per 100,000, and the age-standardized DALYs rate was 301.58 (95% UI: 255.06, 332.06) per 100,000. Join-point analysis revealed an increasing trend in age-standardized prevalence rates between 1990 and 2021, while mortality and DALYs rates decreased. Decomposition analysis identified changes in epidemiological rates as the primary driver of the global increase in HHD deaths and DALYs over this period. Metabolic risks, particularly high systolic blood pressure, were significant contributors to HHD-related mortality and DALYs, with a pronounced impact in high-SDI regions. Regions including the United States and Taiwan (China) exhibited the highest effective disparity, indicating substantial potential for improving HHD management relative to their SDI. Projections using the BAPC model indicate a continued rise in HHD incidence and global deaths by 2050.

**Conclusion:**

Despite declining mortality/DALYs, HHD burden grows in older adults globally. Rising prevalence and projected incidence/deaths demand enhanced prevention. Metabolic risk dominance (high-SDI regions) prioritizes blood pressure control. Marked inequalities [US, Taiwan (China)] reveal opportunities for optimized resource allocation. Findings support tailored strategies to mitigate HHD impact.

## 1 Introduction

Hypertension, defined as systolic/diastolic blood pressure ≥140/90 mm Hg, is a major global risk factor for cardiovascular diseases and a leading cause of DALYs (1–3). The worldwide population is aging rapidly; the proportion of individuals aged 60 and above increased from 9.2% in 1990 to 11.7% in 2013, and is projected to reach 21.1% by 2050 (4). Chronic hypertension, primarily through myocardial fibrosis and left ventricular hypertrophy, drives the development of HHD, substantially elevating the risk of cardiovascular events and mortality (5–8).

Despite its clinical significance, global control of hypertension remains inadequate, with only 32.5% of treated patients achieving target blood pressure levels (9). Modifiable risk factors such as high body mass index (BMI) and high salt intake further compound the disease burden (10). Excessive salt consumption, for instance, adversely affects endothelial function, disrupts neuro-humoral-immune regulation, and increases peripheral resistance, thereby promoting hypertension and HHD progression (11). HHD has also been identified as the second leading cause of heart failure, after ischemic heart disease (12, 13). Hypertension is a prominent global health issue, chiefly responsible for the development of cardiovascular and cerebrovascular ailments (12, 14). Previous studies on HHD burden have been limited in scope, often focusing on specific regions or offering insufficient assessment of temporal trends and risk factor contributions (15–17). A recent GBD study on HHD has also concentrated on this aspect (18). In particular, there is a lack of comprehensive global analyses that integrate socio-demographic disparities and long-term projections. To address these gaps, this study provides an updated assessment of the global burden of HHD from 1990 to 2021 and projects future trends through 2050. We estimate incidence, prevalence, mortality, and DALYs across 204 countries and territories, analyze temporal trends and disparities by SDI, age, sex, and region, and quantify the contributions of major risk factors to HHD-related DALYs. Using data from the GBD 2021 study and applying stratified analyses, decomposition methods, and BAPC modeling, we further project the future burden of HHD and discuss its implications for long-term healthcare planning and policy development.

## 2 Methods

### 2.1 Source

All data for this study were obtained from the GBD 2021 database (https://vizhub.healthdata.org/gbd-results/). This standardized database compiles burden estimates for 371 diseases and 88 risk factors across 204 countries and territories (19). Hypertensive heart disease (HHD) is a constellation of left ventricular (LV) morphological and functional abnormalities that includes LV hypertrophy (LVH) as its hallmark (20). The study specifically extracted data on HHD among individuals aged 65 and older from 1990 to 2021 to assess its prevalence trends and disease burden.

The extracted data include the following dimensions: demographic characteristics (age group: 65+, gender, and year: 1990–2021), geographical classification (204 countries/regions globally, including world health organization (WHO) member states, 5 SDI regions, and 21 GBD regions), and the SDI (21). Based on SDI, the 204 countries/regions were categorized into five development levels: low, lower-middle, middle, upper-middle, and high. Regional classification: The 21 GBD regions were grouped based on epidemiological similarities and geographical proximity (22).

### 2.2 Analysis indicators

We assessed the burden of HHD using three core metrics. Prevalence was defined as the number of HHD cases within the specified population. Mortality was defined as the number of deaths attributable to HHD. Disability-adjusted life years (DALYs) were defined as a composite measure that combines years of life lost due to premature mortality (YLLs) with years lived with disability (YLDs) (23).

All estimates are reported as counts and age-standardized rates (per 100,000 population), using the GBD World Standard Population. Each estimate is accompanied by a 95% uncertainty interval (UI), derived from the 2.5th and 97.5th percentiles of 1,000 posterior draws, reflecting uncertainty in input data and modeling choices.

### 2.3 Analysis methods

To further explore trends and regional variation, multiple advanced modeling strategies were applied as outlined below. The global disease burden of HHD among older adults from 1990 to 2021 was assessed using Excel 2021 and R version 4.3.3. Data were cleaned, organized, and analyzed with R packages (dplyr, officer, and ggplot2), considering a *p*-value of < 0.05 as significant. Subjects were divided into seven age groups (65–69, 70–74, 75–79, 80–84, 85–89, 90–94, and 95+) based on GBD 2021 criteria to examine changes in disease burden across ages and sexes. The EAPC was calculated and visualized to evaluate time trends in age-standardized rates across subgroups. If the lower limit of the 95% confidence interval for EAPC is above 0, it suggests an increasing trend in age-standardized rates; below 0 indicates a decreasing trend. The Join-point Regression Model analyzed trends from 1990 to 2021, calculating average annual percentage change (AAPC) and annual percentage change (APC). A stable trend has a 95% confidence interval including 0, while AAPC or APC significantly above or below 0 indicates an increasing or decreasing trend, respectively. Analyses were done using Join-point software (version 4.9.0.0) and visualized using R software. Pearson's correlation was used to assess associations between SDI and age-standardized HHD rates. The study also examined the proportion of age-standardized mortality and DALYs due to HHD linked to high BMI, kidney dysfunction, and metabolic risks.

### 2.4 Decomposition and prediction analysis

To differentiate the impact of demographic transitions from epidemiological shifts, decomposition methods quantified the effects of population growth, aging, and epidemiological shifts on disease burden. The APC model, based on Poisson distribution, assessed the influence of age, period, and cohort factors on disease risk. To simplify parameter estimation, the Bayesian Markov Chain Monte Carlo algorithm was used with the APC model. The model was subsequently applied to forecast HHD burden from 2022 to 2050 (24).

### 2.5 Health inequality analysis

To address multidimensional inequality, this study utilized the Slope Index of Inequality (SII) and the Concentration Index (CII) from the WHO to assess absolute and relative inequalities in disease burden, specifically HHD, across countries and regions (25). Slope Index of Inequality (SII): this is a quantitative indicator that measures the absolute difference in health outcomes across the socioeconomic gradient (such as income, SDI) through regression analysis. A higher SII value indicates greater health inequality between different socioeconomic groups, while values closer to zero suggest more equal health status across social strata. The SII is mainly combined with SDI (socio-demographic index) and geographical regions to analyze the inequality of disease burden (including prevalence and DALYs) and explore the impact of socioeconomic and geographical differences on health inequality. Concentration Index of Inequality (CII): the CII is an indicator that measures the degree of concentration of health outcomes across socioeconomic distributions. The calculation method involves matching the cumulative proportion of DALYs with the cumulative distribution of the population ranked by SDI, and then calculating the area under the Lorenz curve through numerical integration. The CII analysis assesses health inequality between different development levels, geographical regions, age, and gender groups, exploring influencing factors and trends. Together, these metrics quantify disparities in HHD burden across socioeconomic strata and allow comparison of inequality over time and between regions.

### 2.6 Frontier analysis

To operationalize development, frontier analysis is a key quantitative tool in GBD database analysis, useful for examining the link between disease burden and socio-demographic development. It evaluates and enhances health system performance using Data Envelopment Analysis (DEA) and Local Polynomial Regression (LOESS) to determine the minimum achievable DALYs based on development levels (26). This analysis establishes a frontier representing the lowest DALYs possible under each country's or region's SDI conditions. The “Effective Difference” measures the gap from this frontier, indicating the potential to reduce HHD DALYs according to a country's development level. We employed DEA with the Free Disposal Hull method to define a nonlinear boundary for age-standardized death rates (ASDR) from 1990 to 2021. To address uncertainty, 100 bootstrapped samples were generated through random sampling with replacement across all countries and years. The average HHD DALYs for each SDI value were smoothed using LOESS regression, excluding countries with exceptionally high efficiency to avoid skewing the results. This approach identifies the theoretical minimum disease burden achievable at each SDI level, allowing policymakers to identify countries underperforming relative to their development status. The effective difference for each country or region was then calculated using 2021 SDI and ASDR data, with countries below the boundary assigned a zero distance.

## 3 Results

### 3.1 Global burden of HHD in the older adults

To establish baseline epidemiology, the global age-standardized prevalence of HHD increased from 125.44 (95% UI: 98.97, 157.96) per 100,000 in 1990 to 148.32 (95% CI: 117.32, 186.28) per 100,000 in 2021, with an EAPC of 0.56%, indicating an upward trend (Table 1). The global age-standardized mortality rate for HHD decreased from 20.92 (95% UI: 17.14, 23.21) per 100,000 in 1990 to 16.32 (95% UI: 13.76, 18.01) per 100,000 in 2021, with an EAPC of −0.68%, showing a declining trend (Table 1). The global age-standardized DALYs for HHD decreased from 406.51 (95% UI: 328.94, 452.24) per 100,000 in 1990 to 301.58 (95% UI: 255.06, 332.06) per 100,000 in 2021, with an EAPC of −0.90%, reflecting a reduction in disease burden (Table 1). To uncover gender disparities, women exhibited higher total HHD prevalence than men (2021:6.803 million vs. 5.702 million), though age-standardized rates showed minimal gender differences (148.86 vs. 146.65 per 100,000). A pronounced disparity emerged in South Asia, where women's age-standardized prevalence significantly exceeded men's (141.39 vs. 77.08 per 100,000; Supplementary Tables S1–S3), potentially linked to pregnancy-related hypertension and unequal healthcare access.

**Table 1:** Age-standardized gout burden results for the global population, five SDI regions, and 21 GBD regions.

**Location**	**Prevalence**			**Death rate**			**DALYs**		
	**1990 (per 100,000 population, 95% UI)**	**2021 (per 100,000 population, 95% UI)**	**EAPCs (95% CI)**	**1990 (per 100,000 population 95% UI)**	**2021 (per 100,000 population, 95% UI)**	**EAPCs (95% CI)**	**1990 (per 100,000 population, 95% UI)**	**2021 (per 100,000 population, 95% UI)**	**EAPCs (95% CI)**
Global	125.44 (98.97, 157.96)	148.32 (117.32, 186.28)	0.56 (0.52, 0.60)	20.92 (17.14, 23.21)	16.32 (13.76, 18.01)	−0.68 (−0.77, −0.58)	406.51 (328.94, 452.24)	301.58 (255.06, 332.06)	−0.90 (−0.99, −0.80)
**SDI**									
High SDI	77.16 (59.92, 98.08)	113.36 (88.38, 139.86)	1.57 (1.45, 1.69)	8.56 (7.74, 9.01)	7.70 (6.54, 8.53)	0.11 (−0.07, 0.29)	155.64 (145.29, 162.29)	149.35 (134.50, 162.92)	0.37 (0.15, 0.58)
High-middle SDI	107.70 (83.52, 137.23)	139.85 (108.60, 177.78)	0.87 (0.83, 0.92)	17.83 (15.57, 19.73)	14.60 (12.53, 16.52)	−0.41 (−0.52, −0.29)	315.23 (278.30, 350.96)	228.02 (201.54, 258.50)	−0.93 (−1.03, −0.83)
Middle SDI	189.85 (150.56, 235.42)	178.07 (140.42, 225.70)	−0.35 (−0.47, −0.22)	35.19 (25.55, 39.43)	20.26 (15.30, 23.96)	−1.76 (−1.98, −1.53)	645.06 (459.80, 729.11)	355.67 (273.61, 414.56)	−1.92 (−2.15, −1.70)
Low-middle SDI	145.51 (115.15, 181.68)	151.46 (118.98, 192.39)	0.08 (0.07, 0.10)	28.04 (21.09, 33.87)	23.15 (19.06, 26.62)	−0.56 (−0.61, −0.51)	545.44 (390.96, 652.79)	438.48 (364.14, 503.27)	−0.69 (−0.73, −0.65)
Low SDI	202.28 (158.28, 263.13)	208.42 (162.49, 268.79)	0.12 (0.09, 0.14)	40.54 (28.07, 50.81)	33.58 (24.66, 40.58)	−0.62 (−0.71, −0.52)	817.25 (545.80, 1035.65)	640.71 (451.46, 786.79)	−0.86 (−0.94, −0.78)
**GBD 21 regions**									
Andean Latin America	150.25 (118.58, 191.62)	148.40 (112.87, 192.67)	0.17 (0.07, 0.28)	14.45 (12.57, 16.33)	8.43 (6.70, 10.38)	−1.17 (−1.56, −0.79)	267.14 (230.82, 301.79)	150.96 (122.10, 184.68)	−1.30 (−1.70, −0.90)
Australasia	31.38 (24.63, 40.13)	52.42 (42.04, 64.39)	1.94 (1.82, 2.06)	3.45 (3.05, 3.70)	2.35 (1.96, 2.59)	−1.04 (−1.46, −0.63)	56.26 (51.83, 59.29)	38.76 (34.46, 42.03)	−1.07 (−1.52, −0.62)
Caribbean	141.51 (112.49, 179.64)	195.46 (151.05, 253.29)	1.24 (1.17, 1.30)	20.24 (17.29, 23.27)	19.72 (16.59, 22.99)	0.40 (0.18, 0.62)	411.49 (340.51, 485.41)	408.76 (340.51, 486.35)	0.36 (0.17, 0.55)
Central Asia	80.12 (57.79, 107.58)	95.57 (64.57, 132.61)	0.95 (0.73, 1.17)	16.04 (13.85, 18.83)	20.62 (17.75, 23.92)	1.31 (0.70, 1.92)	323.93 (284.08, 368.10)	376.11 (320.94, 441.92)	0.67 (0.05, 1.29)
Central Europe	100.27 (73.65, 131.25)	145.99 (107.10, 186.69)	1.70 (1.54, 1.85)	23.30 (21.94, 24.53)	25.38 (22.89, 27.31)	0.84 (0.58, 1.11)	413.11 (394.83, 432.64)	418.09 (380.52, 451.92)	0.56 (0.31, 0.81)
Central Latin America	136.82 (107.98, 171.60)	118.99 (92.15, 153.95)	−0.65 (−0.72, −0.59)	18.63 (17.40, 19.38)	9.63 (7.97, 11.13)	−2.21 (−2.41, −2.00)	323.85 (308.78, 335.84)	165.67 (141.03, 193.20)	−2.29 (−2.53, −2.05)
Central Sub-Saharan Africa	221.58 (161.47, 300.53)	239.11 (176.86, 320.43)	0.28 (0.22, 0.34)	67.53 (40.70, 91.84)	66.29 (42.22, 92.93)	−0.09 (−0.14, −0.04)	1326.85 (764.06, 1844.61)	1213.12 (774.02, 1694.91)	−0.34 (−0.40, −0.28)
East Asia	216.66 (168.44, 272.05)	193.16 (147.41, 245.89)	−0.63 (−0.86, −0.39)	41.86 (30.02, 48.57)	18.73 (13.00, 24.13)	−2.63 (−3.01, −2.25)	704.81 (483.22, 825.16)	292.61 (210.37, 371.85)	−2.90 (−3.30, −2.50)
Eastern Europe	28.67 (21.29, 38.82)	42.80 (29.82, 58.32)	1.73 (1.55, 1.92)	4.48 (4.25, 4.70)	7.41 (6.68, 8.04)	1.71 (0.78, 2.66)	101.62 (96.24, 106.63)	139.74 (128.15, 152.79)	0.86 (−0.10, 1.82)
Eastern Sub-Saharan Africa	282.19 (216.06, 371.20)	291.80 (224.00, 375.41)	0.08 (0.06, 0.10)	58.45 (39.64, 72.82)	42.56 (29.44, 54.66)	−1.14 (−1.21, −1.07)	1151.61 (741.13, 1457.21)	786.16 (549.70, 992.18)	−1.39 (−1.48, −1.31)
High-income Asia Pacific	57.42 (41.74, 75.01)	57.56 (43.76, 72.35)	−0.21 (−0.41, −0.01)	10.54 (9.05, 11.39)	2.97 (2.34, 3.47)	−3.66 (−4.41, −2.89)	159.13 (141.74, 169.77)	47.14 (40.22, 56.34)	−3.63 (−4.33, −2.92)
High-income North America	91.78 (70.26, 118.35)	150.67 (119.50, 181.92)	1.93 (1.78, 2.07)	6.93 (6.29, 7.27)	10.41 (9.06, 11.52)	1.51 (1.33, 1.70)	152.14 (144.01, 157.76)	236.49 (214.90, 259.50)	1.81 (1.64, 1.98)
North Africa and Middle East	239.48 (192.81, 299.16)	243.27 (192.08, 303.63)	0.13 (0.08, 0.18)	56.89 (43.87, 68.05)	39.54 (31.48, 46.22)	−1.04 (−1.20, −0.88)	1025.93 (781.23, 1223.48)	692.12 (548.96, 810.45)	−1.18 (−1.31, −1.05)
Oceania	115.73 (90.24, 147.95)	106.90 (83.70, 136.78)	−0.36 (−0.42, −0.31)	26.11 (17.29, 34.39)	18.97 (13.06, 26.88)	−1.11 (−1.17, −1.06)	583.65 (371.23, 789.67)	434.63 (296.51, 627.55)	−1.02 (−1.07, −0.97)
South Asia	104.13 (82.67, 129.59)	111.14 (86.64, 142.69)	0.19 (0.17, 0.21)	17.92 (11.73, 24.22)	16.54 (13.11, 21.44)	−0.17 (−0.30, −0.03)	345.08 (226.27, 463.85)	301.71 (238.05, 392.30)	−0.40 (−0.49, −0.31)
Southeast Asia	168.08 (135.87, 207.43)	163.33 (131.20, 205.18)	−0.19 (−0.23, −0.14)	29.57 (20.74, 35.80)	22.92 (16.89, 26.49)	−0.82 (−0.87, −0.77)	611.53 (426.61, 743.70)	464.82 (339.68, 536.92)	−0.89 (−0.93, −0.85)
Southern Latin America	88.10 (64.94, 117.81)	110.10 (78.29, 145.84)	0.98 (0.92, 1.04)	16.29 (15.14, 17.08)	13.77 (12.01, 14.78)	−0.16 (−0.33, 0.01)	289.43 (274.80, 301.39)	213.17 (193.34, 226.44)	−0.67 (−0.81, −0.53)
Southern Sub–Saharan Africa	196.79 (147.15, 256.08)	210.62 (157.38, 272.23)	0.25 (0.19, 0.30)	38.20 (32.67, 49.17)	47.42 (41.51, 55.21)	0.84 (0.39, 1.30)	758.91 (665.03, 944.82)	889.11 (785.13, 1034.16)	0.66 (0.20, 1.12)
Tropical Latin America	149.93 (118.51, 187.39)	166.01 (129.09, 213.56)	0.32 (0.29, 0.36)	23.10 (21.23, 24.19)	12.39 (10.80, 13.52)	−1.81 (−1.94, −1.68)	455.00 (431.30, 470.25)	234.81 (213.32, 255.22)	−2.04 (−2.17, −1.91)
Western Europe	65.79 (49.45, 86.39)	100.84 (77.96, 128.11)	2.02 (1.70, 2.34)	8.66 (7.77, 9.16)	8.21 (6.66, 9.07)	0.40 (0.21, 0.60)	134.86 (124.63, 141.19)	111.10 (95.08, 120.73)	−0.05 (−0.22, 0.12)
Western Sub-Saharan Africa	257.43 (201.82, 331.62)	266.25 (206.68, 342.16)	0.15 (0.10, 0.19)	34.57 (25.65, 43.67)	28.78 (18.85, 34.87)	−0.77 (−0.93, −0.61)	712.75 (522.26, 889.54)	590.83 (378.81, 726.06)	−0.78 (−0.93, −0.62)

### 3.2 Regional burden of HHD in the older adults

Mapping development gradients, the prevalence in high SDI regions increased significantly from 77.16 (95% UI: 59.92, 98.08) per 100,000 in 1990 to 113.36 (95% UI: 88.38, 139.86) per 100,000 in 2021 in the five SDI regions, with an EAPC of 1.57 (Table 1). The mortality rate in high SDI regions decreased slightly from 8.56 (95% UI: 7.74, 9.01) per 100,000 in 1990 to 7.70 (95% UI: 6.54, 8.53) per 100,000 in 2021, with an EAPC of 0.11 (Table 1). DALYs in high SDI regions decreased slightly from 155.64 (95% UI: 145.29, 162.29) per 100,000 in 1990 to 149.35 (95% UI: 134.50, 162.92) per 100,000 in 2021, with an EAPC of 0.37 (Table 1). This indicates that while the prevalence of HHD continues to rise in high SDI regions, the mortality rate and health burden are effectively controlled due to better healthcare and disease management.

To identify progress zones, in moderate SDI regions showed that the prevalence decreased from 189.85 (95% UI: 150.56, 235.42) per 100,000 in 1990 to 178.07 (95% UI: 140.42, 225.70) per 100,000 in 2021, with an EAPC of −0.35 (Table 1). Moderate SDI regions demonstrated substantial progress: between 1990 and 2021, mortality plummeted from 35.19 (95% UI: 25.55–39.43) to 20.26 (15.30–23.96) per 100,000, while DALYs fell from 645.06 (459.80–729.11) to 355.67 (273.61–414.56) per 100,000. This decline (EAPCs: −1.76 mortality; −1.92 DALYs; Table 1) reflects improved healthcare. In low SDI regions, prevalence remained persistently high (1990: 202.28; 2021: 208.42 per 100,000; EAPC +0.12). Despite modest reductions in mortality (40.54 to 33.58; EAPC −0.62) and DALYs (817.25 to 640.71; EAPC −0.86), the burden remains severe—likely due to suboptimal treatment and health management.

**Figure 1:**
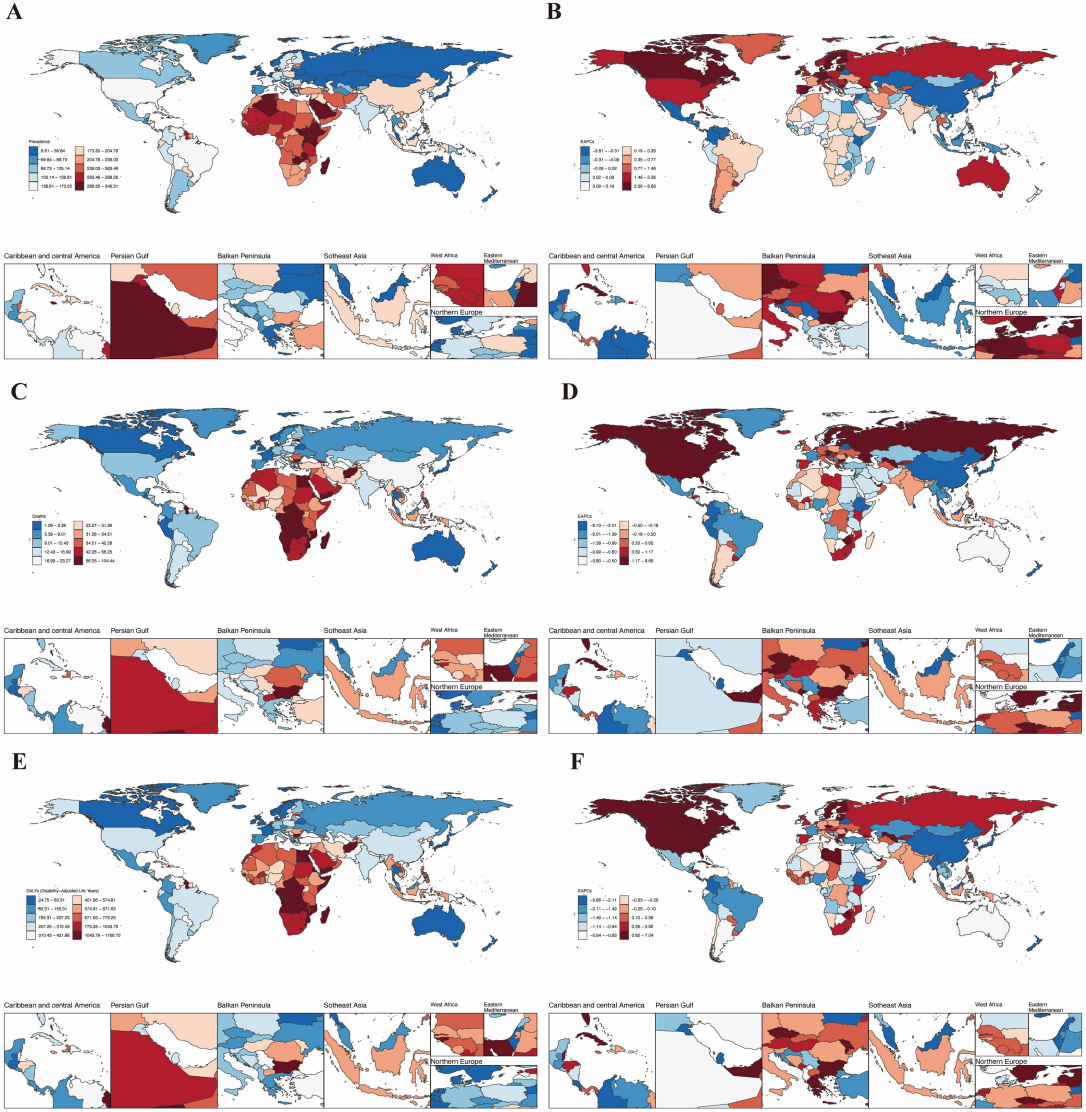
The global burden of HHD across 204 countries and regions in 2021. **(A)** Age-standardized prevalence; **(B)** age-standardized mortality rate; **(C)** age-standardized disability-adjusted life years (DALYs); **(D)** age-standardized prevalence of the estimated annual percentage change (EAPC); **(E)** age-standardized mortality rate of the EAPC; **(F)** Age-standardized DALYs of the EAPC.

In the 21 GBD regions, all indicators in Andean Latin America remained stable or showed a downward trend (Table 1, Figure 1). In East Asia, while the age-standardized prevalence rate increased significantly (EAPC = 1.57, 95% UI: 1.45–1.69), the rising case volume is primarily driven by population aging, alongside contributions from lifestyle changes. The total number of cases in 2021 reached 2.436 million, nearly doubling from 846,000 in 1990. The total number of cases increased from 1.55 million in 1990 to 4.066 million in 2021, but the age-standardized prevalence rate showed an EAPC of −0.63 (−0.86 to −0.39), suggesting that population growth and aging are the main factors driving the absolute increase, while the age-adjusted prevalence rate actually decreased. DALYs in East Asia decreased significantly (EAPC −2.90%), reflecting that despite the increase in incidence, overall disease management and health conditions have improved (Table 1). In Sub-Saharan Africa, the age-standardized prevalence rates in East, Central, and West Africa were the highest globally (291.80–266.25 per 100,000), but the EAPC was close to zero, indicating that the disease burden remains high and has not improved (Table 1).

### 3.3 National and regional burden of HHD in the older adults

We next assessed that Jordan recorded the highest older adults age-standardized prevalence (341.89 per 100,000; Figure 1A, Table 1), while Afghanistan had the worst mortality (78.26 per 100,000; Figure 1B). Bulgaria bore the highest DALYs burden (1,739.30 per 100,000; Figure 1C). Conversely, Belgium showed the lowest prevalence (23.01 per 100,000; Figure 1A). Belarus had the lowest age-standardized mortality rate and DALYs for HHD in the older adults, with rates of 1.10 (95% UI: 0.93, 1.33) per 100,000 and 25.00 (95% UI: 20.70, 31.05) per 100,000, respectively (see Figures 1B, C).

Longitudinal trend analysis showed that the age-standardized burden of HHD declined in Belarus, Colombia, and Guam. Belarus demonstrated the most substantial declines across all metrics: age-standardized prevalence (EAPC = −3.77, 95% UI: −4.18, −3.35; Table 1), age-standardized mortality (EAPC = −5.67, 95% UI: −6.78, −4.55; Table 1), and age-standardized DALYs (EAPC = −5.80, 95% UI: −6.95, −4.63; Table 1). In contrast, areas like Moldova experienced rising case volumes despite stable or declining age-adjusted prevalence rates, highlighting population aging as the dominant factor. Conversely, the Republic of Moldova, Estonia, and Latvia exhibited an increasing trend in the burden of HHD. Latvia showed the most significant increase in age-standardized prevalence (EAPC = 6.76, 95% UI: 6.13, 7.40) and DALYs (EAPC = 6.97, 95% UI: 5.77, 8.18). Additionally, the burden of HHD in the older adults showed an upward trend in other countries and regions (Figures 1D–F). Detailed disease burden data for 204 countries and regions can be found in Supplementary Tables S1–S4.

### 3.4 Age-sex-time association analysis of HHD in the older adults

**Figure 2:**
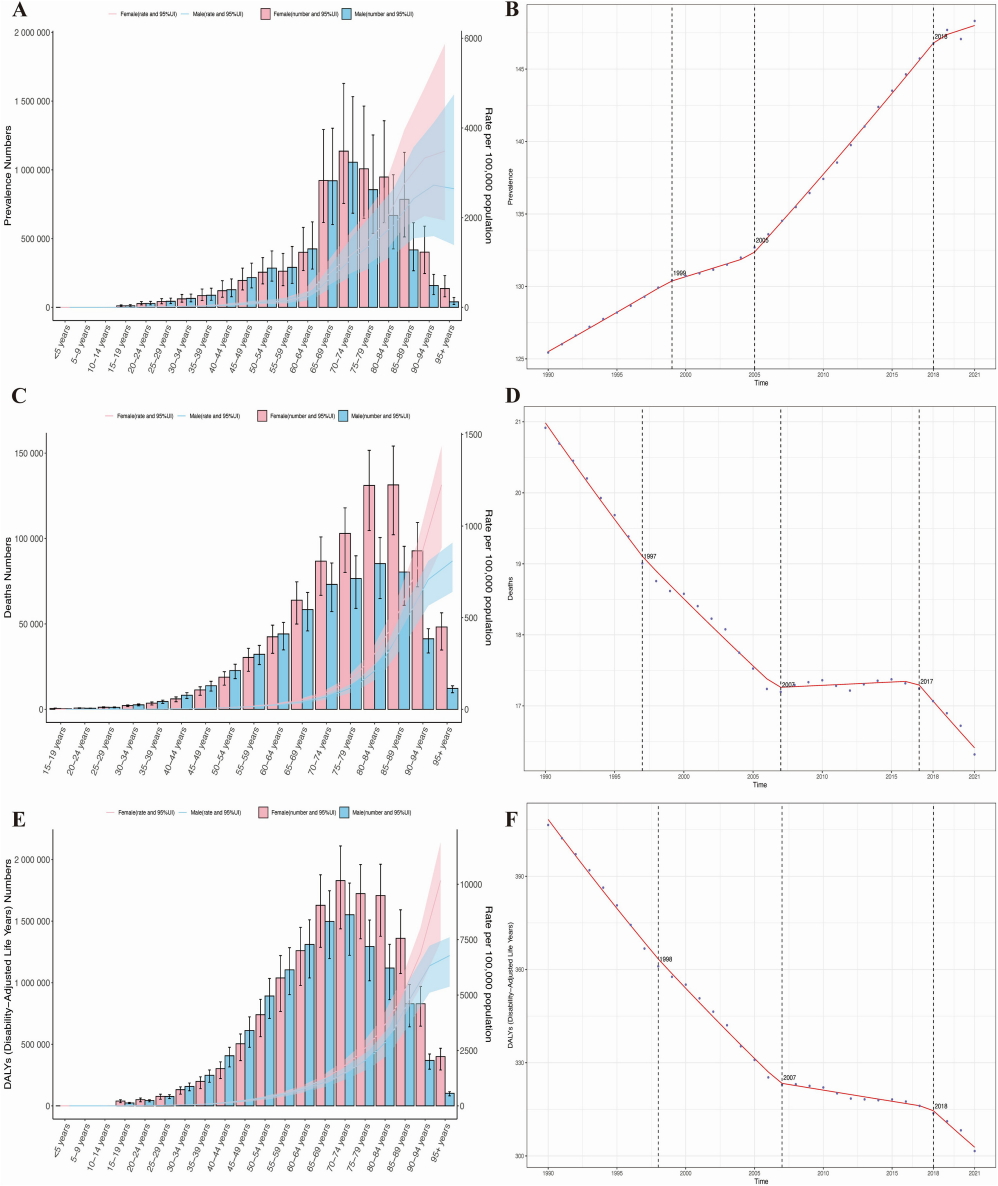
Age- and sex-specific trends in the burden of HHD and the results of Joinpoint regression analysis. **(A)** Prevalence; **(B)** mortality rate; **(C)** disability-adjusted life years (DALYs); **(D)** joinpoint analysis of prevalence; **(E)** joinpoint analysis of mortality rate; **(F)** joinpoint analysis of DALYs.

Trends by sex and age group revealed that in 2021, the age-standardized prevalence, mortality, and DALYs of HHD in individuals aged 65 and older increased with age. However, this increase was not significant, and there was no notable difference in the disease burden between older men and women (Figures 2A–C). Age-time association analysis reveals that global age-standardized HHD prevalence, mortality, and DALYs remained largely unchanged across age groups from 1990 to 2021. However, HHD burden increased significantly with advancing age (Supplementary Figure S1). Sex-time association analysis indicates that globally, the age-standardized incidence, prevalence, and DALYs of HHD in older adults remained stable in both men and women from 1990 to 2021, with no significant changes over time. Notably, in Western Sub-Saharan Africa and Central Sub-Saharan Africa, the age-standardized prevalence in men is twice that in women, but the age-standardized mortality and DALYs are twice as high in women as in men. In other regions, there were no significant gender differences in disease burden (Supplementary Figure S1).

### 3.5 Trend of changes in the burden of HHD in the older adults

Resolving temporal divergence, joinpoint regression analysis indicates that from 1990 to 2021, the prevalence of HHD in the older adults globally showed an overall increasing trend, while mortality and DALYs exhibited a declining trend. Specifically, the AAPC for prevalence was 0.727 (95% CI: 0.710, 0.743), for mortality was −0.147 (95% CI: −0.152, −0.142), and for DALYs was −3.411 (95% CI: −3.493, −3.329), with varying rates of change. Significant changes in all three disease burden indicators occurred primarily in the years 1990, 2007 (for prevalence in 2007), and 2018 (for mortality in 2017), as shown in Figures 2D–F. Since 2010, the downward trend in disease burden observed in 2018 has slightly slowed, but it remains in a significant declining phase. The slope of each segment of the curve is detailed in Supplementary Figure S1.

### 3.6 Association between the burden of HHD in the older adults and SDI

**Figure 3:**
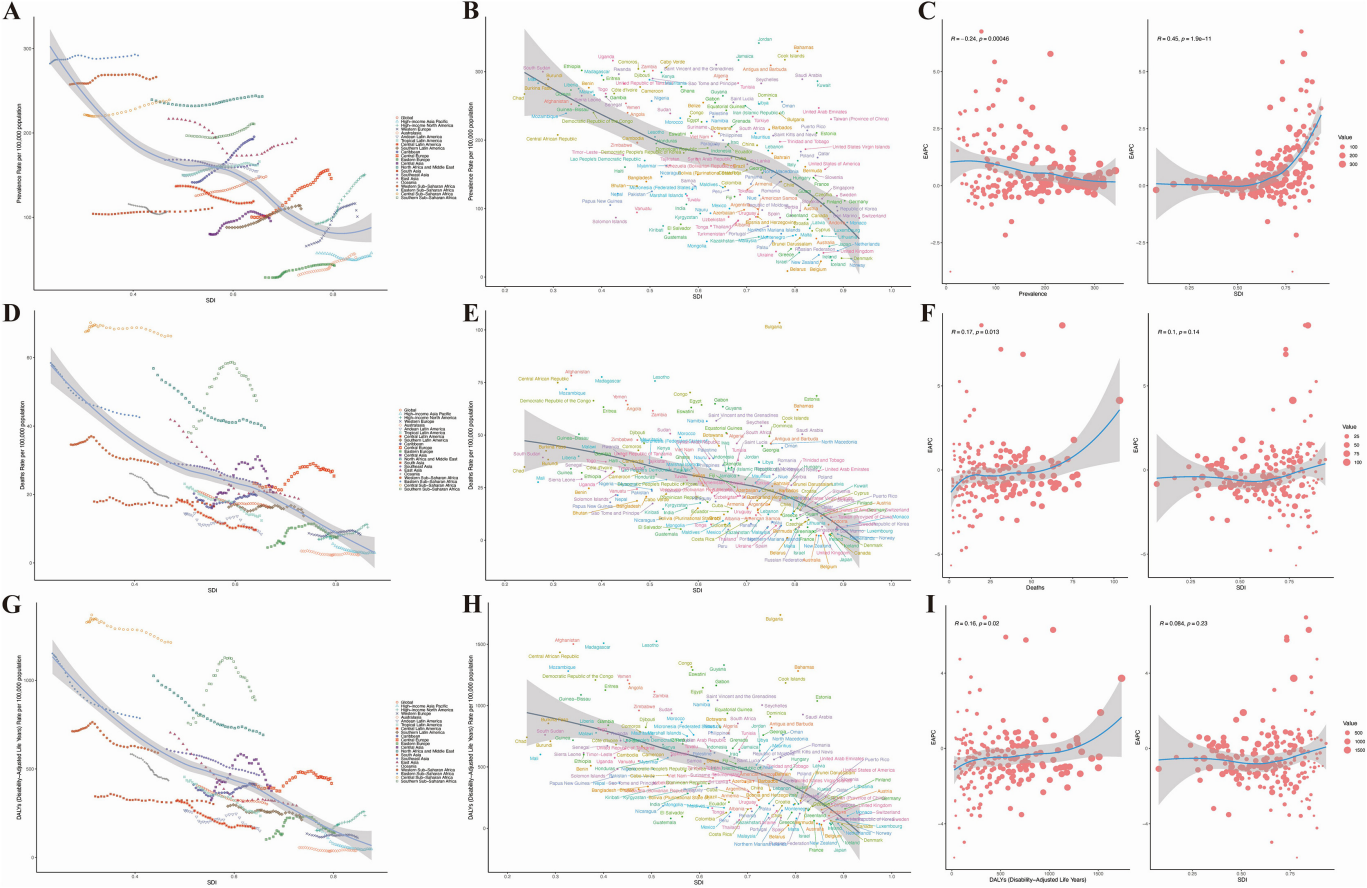
Results of SDI analysis. **(A)** Prevalence in 21 regions; **(B)** mortality rate in 21 regions; **(C)** DALYs in 21 regions; **(D)** prevalence in 204 countries; **(E)** mortality rate in 204 countries; **(F)** DALYs in 204 countries; **(G)** estimated annual percentage change (EAPC) in prevalence; **(H)** EAPC in mortality rate; **(I)** EAPC in DALYs.

To further evaluate the impact of SDI on the HHD burden, our analysis comprehensively reveals that globally and across all 21 GBD regions, a non-linear relationship exists between the Socio-demographic Index (SDI) and age-standardized rates of HHD incidence, prevalence, and DALYs. The fitted curves for the 21 regions and 204 countries show that, overall, the disease burden gradually decreases with increasing SDI. In other words, when SDI is very low, which typically indicates underdeveloped countries, the prevalence is high. As development progresses, the prevalence decreases. Among these regions, Bulgaria exhibits the fastest increase in disease burden, as seen in Figures 3A, D, G. The curves formed by these points across the 21 regions suggest that the level of development, represented by the SDI value, is already established, but over time, as countries become more developed, these curves extend forward. It is also evident that the lines for most regions remain relatively unchanged, meaning that while development continues, the reduction in disease burden is minimal, and in many regions, it even increases.

In the 204 countries, a significant correlation between the prevalence of HHD and SDI was found (*p* < 0.001), as shown in Figures 3B, E, H. Interestingly, as prevalence increases, the EAPC remains relatively unchanged (Figure 3C). However, as SDI rises, the EAPC increases, suggesting that higher levels of economic development are associated with a gradual increase in disease burden. For mortality and DALYs associated with HHD, the EAPC also increases, but as SDI increases, the EAPC tends to stabilize (Figures 3F, I). This indicates that in countries with higher SDI, the burden of HHD is becoming more severe, as shown in Figures 3G, H, I.

### 3.7 Age-Period-Cohort analysis results of HHD burden in the older adults

**Figure 4:**
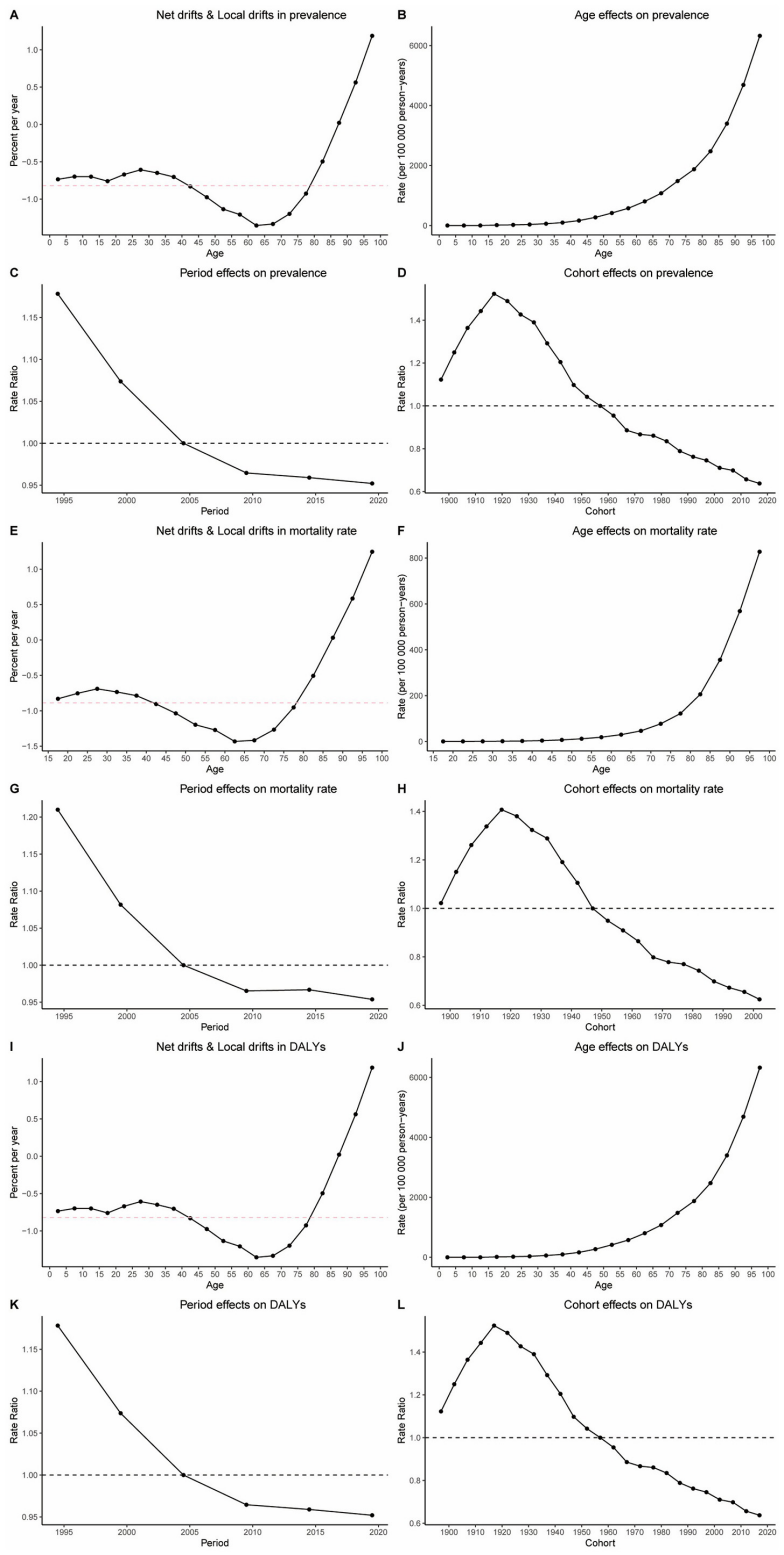
Results of age-period cohort analysis. **(A)** Net drifts and local drifts in prevalence; **(B)** age effect on prevalence; **(C)** period effect on prevalence; **(D)** cohort effect on prevalence; **(E)** net drifts and local drifts in mortality rate; **(F)** age effect on mortality rate; **(G)** period effect on mortality rate; **(H)** cohort effect on mortality rate; **(I)** net drifts and local drifts in DALYs; **(J)** age effect on DALYs; **(K)** period effect on DALYs; **(L)** cohort effect on DALYs.

When disentangling etiological factors, age-period-cohort analyses of HHD burden (prevalence, mortality, DALYs) show consistent trends in older adults. Specifically, age effect analysis indicates a significant increase in the disease burden of HHD as age increases, as seen in Figures 4B–D. Period effect analysis shows that from 1990 to 2021, the disease burden of HHD gradually increased over time, as shown in Figures 4F–H. Cohort effect analysis reveals that more recently born cohorts have a significantly higher disease burden of HHD compared to earlier born cohorts, as seen in Figures 4J–L. Additionally, we used net and local drift (Figure 4A) to further validate the dynamic change of disease risk with age. This section illustrates the annual average trend of prevalence changes over time for different age groups. The results show that as age increases, the disease burden of HHD further intensifies (Figures 4A, E, I).

### 3.8 Decomposition analysis results of the disease burden of HHD in the older adults

**Figure 5:**
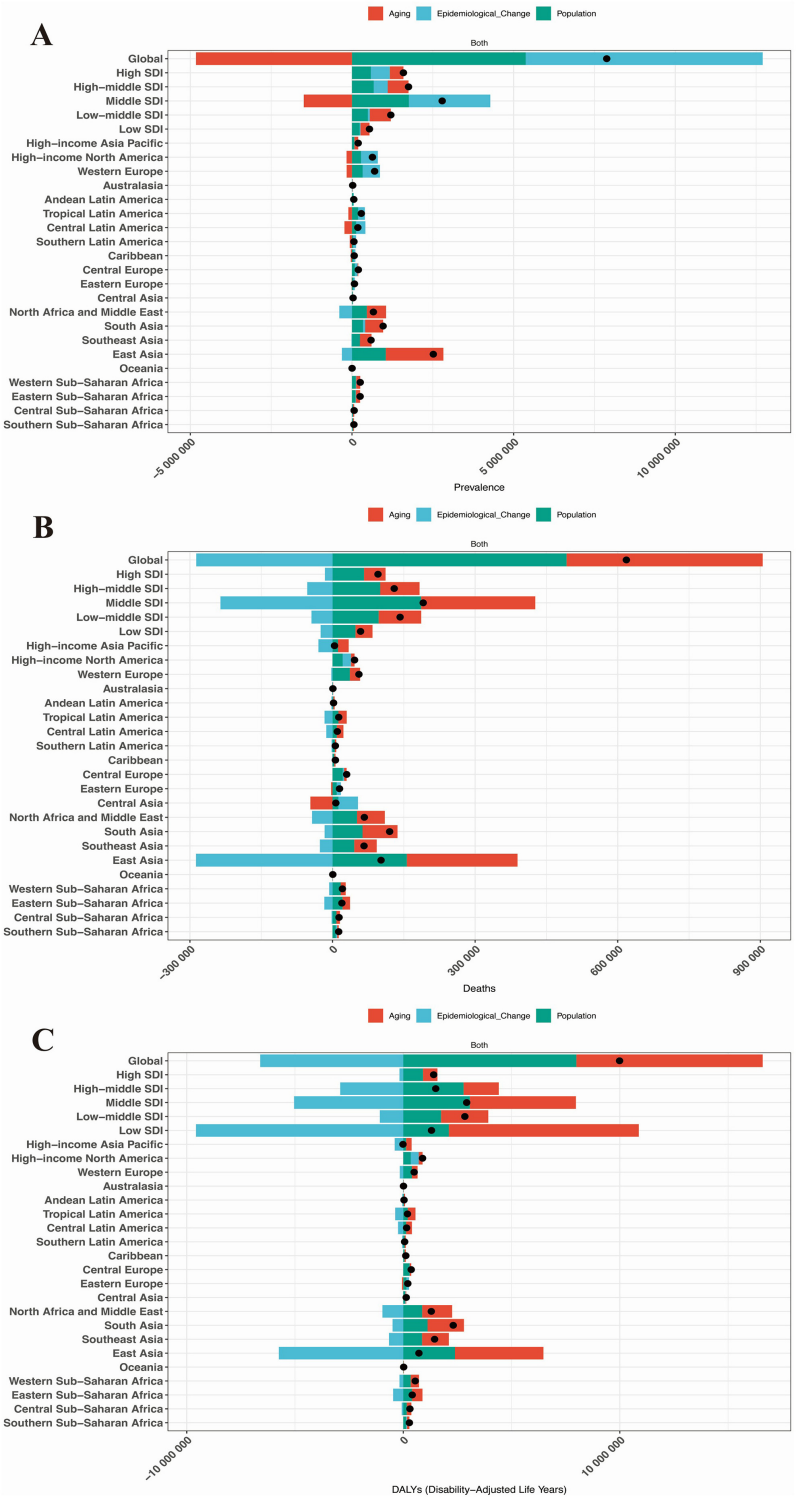
Results of decomposition analysis. **(A)** Prevalence in the global population, 5 SDI regions, and 21 GBD regions; **(B)** Mortality rate in the global population, 5 SDI regions, and 21 GBD regions; **(C)** DALYs in the global population, 5 SDI regions, and 21 GBD regions.

To disentangle the contributions of demographic transitions from epidemiological shifts, decomposition analysis reveals that aging primarily drives the HHD disease burden in terms of prevalence globally, across five SDI regions, and in 21 GBD regions, whereas epidemiological changes dominate mortality and DALYs burdens (Figures 5A–C). Specifically, these three factors have no contribution to the disease burden of HHD in Australasia, Andean Latin America, and Oceania. In all other regions, population growth, aging, and epidemiological changes all contributed to the increase in the disease burden of HHD, with epidemiological changes being the primary factor responsible for the worsening of the disease burden, as shown in Figures 5A–C.

### 3.9 Prediction analysis results of the disease burden of HHD in the older adults

**Figure 6:**
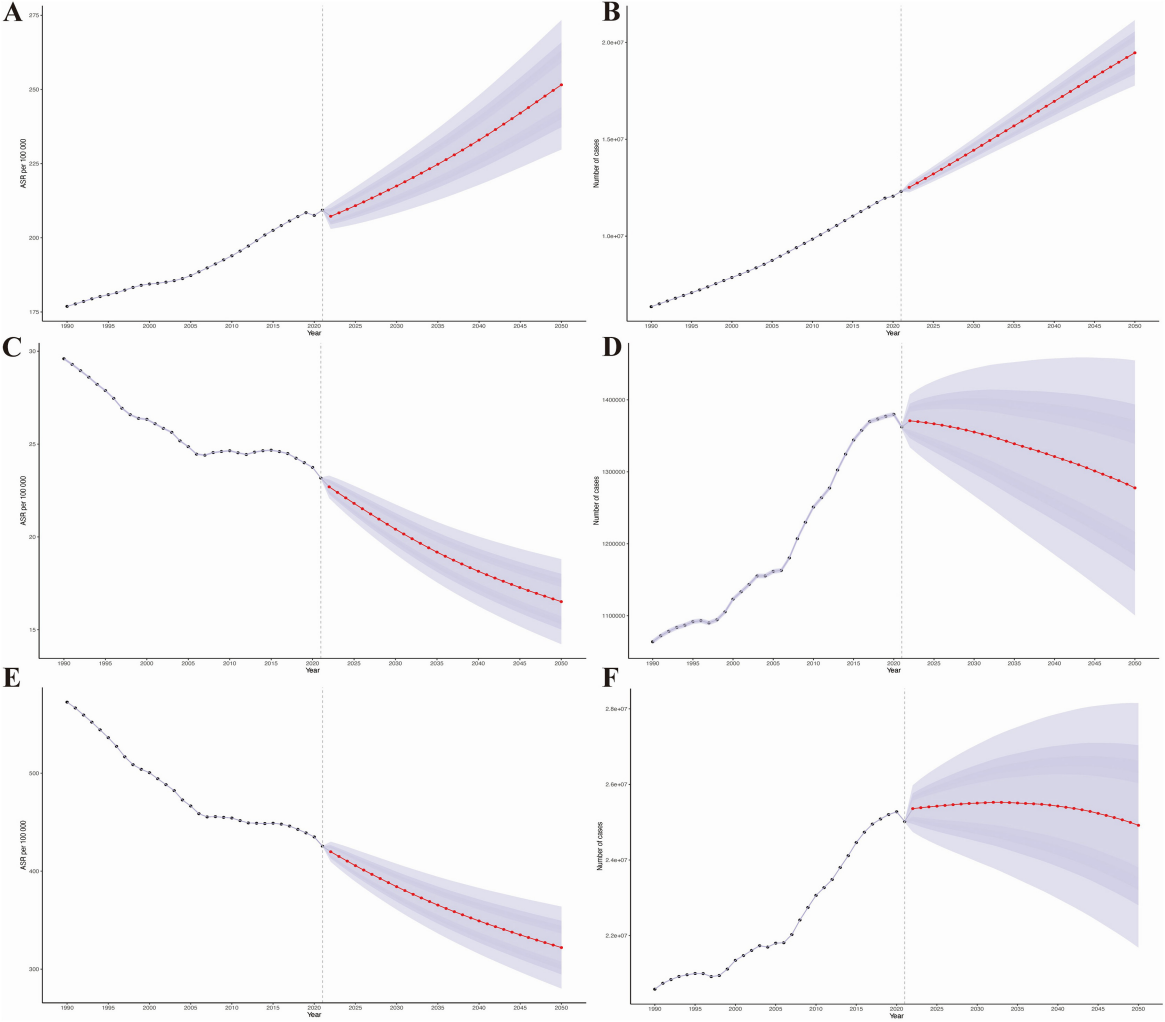
Results of predictive analysis. **(A)** Age-standardized prevalence; **(B)** Age-standardized mortality rate; **(C)** Age-standardized disability-adjusted life years (DALYs); **(D)** Actual prevalence; **(E)** Actual mortality rate; **(F)** Actual DALYs.

Subsequently, the prediction analysis shows that from 2022 to 2050, the disease burden of HHD in older adults will further increase globally (Figures 6A–F). By 2050, the age-standardized prevalence, mortality, and DALYs of HHD in the older adults will increase to 251.56 (95% CI: 229.77, 273.36) per 100,000, 16.51 (95% CI: 14.23, 18.80) per 100,000, and 322.04 (95% CI: 280.25, 363.85) per 100,000, respectively, as detailed in Supplementary Table S3. This means that by 2050, there will be an additional 12,307,531.48 (95% CI: 12,297,828.76, 12,317,234.21) older adults with HHD, 1,362,282.19 (95% CI: 1,359,024.01, 1,365,540.37) older adults deaths due to HHD, and a loss of 25,018,879.31 (95% CI: 25,005,047.40, 25,032,711.22) life years, as detailed in Supplementary Table S3.

Additionally, our prediction analysis for different age groups reveals that the disease burden of HHD will show a significant upward trend across all age groups, with the increase being more pronounced in older age groups (Figures 6A–F).

### 3.10 Health inequality analysis results of HHD in the older adults

**Figure 7:**
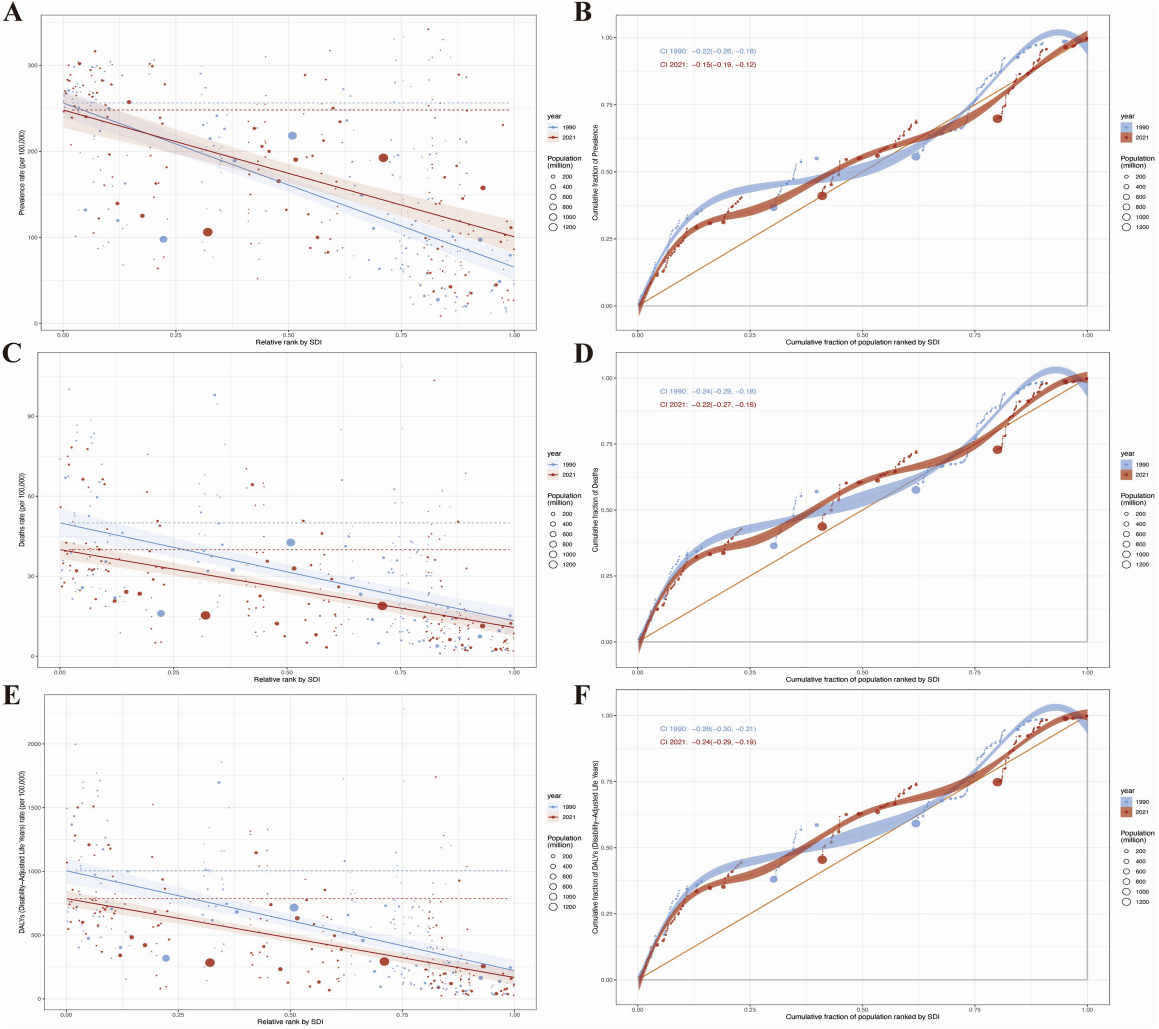
Results of health inequality analysis. **(A)** Trends in the Slope Index of Inequality (SII) for age-standardized prevalence from 1990 to 2021; **(B)** Comparison of absolute income-related health inequality in age-standardized prevalence of HHD between 1990 and 2021; **(C)** Trends in the SII for age-standardized mortality rate from 1990 to 2021; **(D)** Comparison of absolute income-related health inequality in age-standardized mortality rate of HHD between 1990 and 2021; **(E)** Trends in the SII for age-standardized DALYs from 1990 to 2021; **(F)** Comparison of absolute income-related health inequality in age-standardized DALYs of HHD between 1990 and 2021.

To quantify inequities, we identified substantial absolute and relative inequalities in HHD associated with the SDI, with a higher burden in low-SDI regions (Figures 7A–F). The health inequality index revealed a reduction in the prevalence gap between the highest and lowest SDI countries, decreasing from −190.74 (95% CI: −217.51, −163.98) in 1990 to −147.29 (95% CI: −177.82, −116.76) in 2021 (Figure 7A). The concentration index for HHD prevalence dropped from −0.22 (95% CI: −0.26, −0.18) in 1990 to −0.15 (95% CI: −0.19, −0.12) in 2021 (Figure 7B).

Regarding mortality, the health inequality index showed a decline in the gap between the highest and lowest SDI regions, from −36.69 (95% CI: −44.31, −29.07) in 1990 to −29.22 (95% CI: −35.39, −23.05) in 2021 (Figure 7C), while the concentration index decreased from −0.24 (95% CI: −0.29, −0.18) in 1990 to −0.22 (95% CI: −0.27, −0.16) in 2021 (Figure 7D).

For DALYs, the disparity between the highest and lowest SDI countries narrowed from −783.66 (95% CI: −930.04, −637.28) in 2019 to −618.94 (95% CI: −730.26, −507.62) in 2021 (Figure 7E), with the concentration index decreasing from −0.26 (95% CI: −0.30, −0.21) in 2019 to −0.24 (95% CI: −0.29, −0.19) in 2021 (Figure 7F).

### 3.11 Frontier analysis results of HHD in the older adults

**Figure 8:**
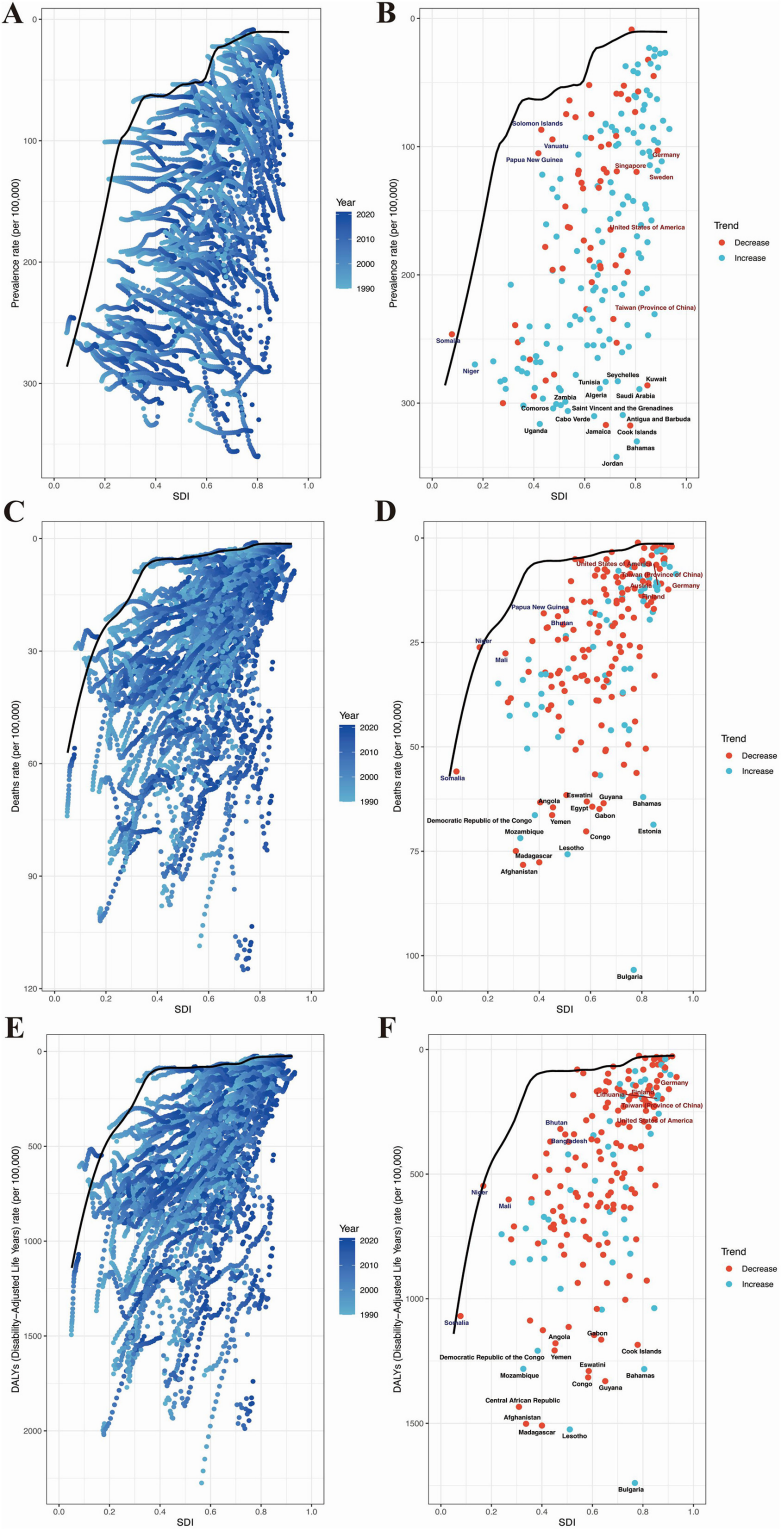
Results of frontier analysis. Panels **(A, C, E)** each point represents a country, with the line indicating the frontier (the best-performing line, representing the least burden of disease). The color of the points ranges from light blue to dark blue, reflecting the change in disease burden from 1990 to 2021. The effective distance of a country from the frontier is defined as the gap between its observed and potentially achievable disease burden. Panels **(B, D, F)** Points with black outlines represent the 15 countries or regions with the largest differences from the frontier. Points with blue outlines represent the 5 low-SDI countries (<0.50) with the smallest differences from the frontier. Points with red outlines represent the 5 high-SDI countries (>0.85) with the largest differences from the frontier. Red points indicate that the disease burden in these countries is decreasing, while blue points indicate that the disease burden is increasing. **(A)** Frontier analysis of the changing trends in age-standardized prevalence from 2019 to 2021. **(B)** Frontier analysis of age-standardized prevalence based on SDI and countries in 1990. **(C)** Frontier analysis of the changing trends in age-standardized mortality rate from 2019 to 2021. **(D)** Frontier analysis of age-standardized mortality rate based on SDI and countries in 1990. **(E)** Frontier analysis of the changing trends in age-standardized DALYs from 2019 to 2021. **(F)** Frontier analysis of age-standardized DALYs based on SDI and countries in 1990.

To understand the potential improvements in the prevalence of HHD based on a country's development status, the effective difference is lowest in high SDI countries; however, countries with intermediate SDI values have the largest effective differences (Figures 8A, C, E). Based on data from 1990 to 2021 and considering age-standardized prevalence and SDI, frontier analysis was conducted to explore the potential for improvement in age-standardized prevalence of HHD considering the level of development of countries and regions. The 15 countries and regions with the largest actual disparities and opportunities for improvement (effective difference range:1,225.01–449.98) consist of Seychelles, Tunisia, Kuwait, Zambia, Algeria, Saudi Arabia, Comoros, Saint Vincent and the Grenadines, Cabo Verde, Antigua and Barbuda, Uganda, Jamaica, Cook Islands, Bahamas, and Jordan (Figure 8B). In low SDI countries (< 0.50), the five countries with the smallest frontier differences for age-standardized mortality are Solomon Islands, Papua New Guinea, Vanuatu, Somalia, and Niger, which are frontier countries (Figure 8B). In high SDI countries (>0.85), the five countries with the largest frontier differences for age-standardized mortality include Germany, Singapore, Sweden, the United States of America, and Taiwan (Province of China) (Figure 8B). The frontier analysis highlights the potential for reducing the burden of HHD across different countries and regions. Despite resource constraints, certain low SDI countries and regions, such as the Solomon Islands, have demonstrated notable success in managing the disease.

In addition, we calculated the top 15 countries with the largest frontier differences in age-standardized mortality rates. These countries include Solomon Islands, Papua New Guinea, Vanuatu, Somalia, Niger, Germany, Singapore, Sweden, the United States of America, Taiwan (Province of China), Eswatini, Guyana, Angola, Egypt, Gabon, Democratic Republic of the Congo, Yemen, Mozambique, Congo, Lesotho, Madagascar, Afghanistan, Bahamas, and Estonia (Figure 8D). In low SDI countries (< 0.50), the five countries with the smallest frontier differences for age-standardized mortality are Papua New Guinea, Bhutan, Mali, Niger, and Somalia (Figure 8D). In high SDI countries (>0.85), the five countries with the largest frontier differences for age-standardized mortality are the United States of America, Taiwan (Province of China), Austria, Germany, and Finland. These countries have underperformed relative to their SDI levels (Figure 8D).

Furthermore, based on data from 1990 to 2021 and using ASDR rates and SDI, frontier analysis results showed that the top 15 countries with the largest frontier differences were Gabon, Angola, Cook Islands, Democratic Republic of the Congo, Yemen, Mozambique, Central African Republic, Afghanistan, Congo, Eswatini, Guyana, Bahamas, Madagascar, and Lesotho (Figure 8F). In low SDI countries (< 0.50), the five countries with the smallest frontier differences for age-standardized mortality are Bhutan, Bangladesh, Niger, Mali, and Somalia (Figure 8F). In high SDI countries (>0.85), the five countries with the largest frontier differences for age-standardized mortality are Germany, Lithuania, Finland, Taiwan (Province of China), and the United States of America (Figure 8F).

### 3.12 Risk factors for mortality and DALYs

Currently, only the risk factors for mortality and DALYs of HHD have been reported. We used the population attributable fraction (PAF), also known as the attributable risk proportion, which refers to the proportion of the total incidence or mortality of a disease in a population that is attributed to a specific exposure factor (smoking, poor diet). It reflects how much the incidence or mortality of the disease would decrease if that exposure factor were eliminated.

**Figure 9:**
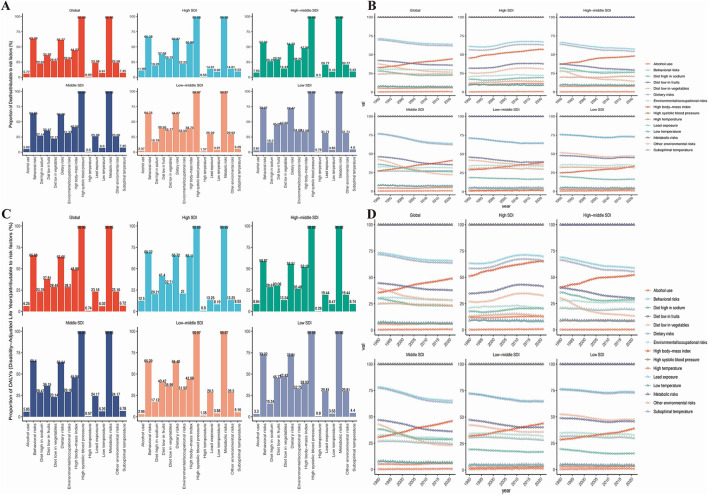
Risk factors for mortality and DALYs in the global population and 5 SDI regions. **(A)** Risk factors for mortality in 2021. **(B)** Trends in the changing risk factors for mortality over time. **(C)** Risk factors for DALYs in 2021. **(D)** Trends in the changing risk factors for DALYs over time.

There are three main risk factors contributing to the mortality and DALYs of HHD: high body-mass index, kidney dysfunction, and metabolic risks. Across the global, five SDI regions, and 21 GBD regions, metabolic risks are the primary risk factor for HHD (Figures 9A, C). Over time, the disease burden attributed to various risk factors has shown an upward trend, with the most noticeable increase being seen in metabolic risks (Figures 9B, D).

## 4 Discussion

This study provides an updated assessment of HHD from 1990 to 2021 at the global, regional, and national levels and examines key demographic and epidemiological drivers. From 1990 to 2021, HHD mortality and DALYs declined, whereas prevalence rose: the age-standardized prevalence increased from 125.44 to 148.32 per 100,000 (average annual change rate 0.56%). Population aging is likely a major driver of this pattern. In 2019, 703 million people aged ≥65 years accounted for 9% of the world's population, a share projected to rise from 10% in 2022 to 16% by 2050—about 1.5 billion older adults (27). Age-related physiological changes—enhanced sodium sensitivity, arterial stiffness, endothelial dysfunction, isolated systolic hypertension, white-coat effect, and orthostatic hypertension—may contribute to higher blood pressure, thereby elevating HHD risk in older adults (32). Thus, population aging expands the pool of biologically susceptible individuals, while shifts in incidence reflect modifiable exposures, particularly high salt intake and physical inactivity (28, 29). Taken together, aging amplifies baseline risk and lifestyle factors modulate incidence, driving a growing HHD burden (30). Geographic differences in these exposures may help explain much of the regional variation in incidence (31, 32).

Despite an increase in prevalence, global mortality rates decreased from 20.92 to 16.32 per 100,000 (with an average annual change of −0.68%), and DALYs declined from 406.51 to 301.58 per 100,000 (−0.90%). These trends are consistent with advancements in early detection, improved treatment modalities, and expanded access to healthcare services (33). However, outcomes varied across the SDI spectrum. In high-SDI regions, mortality rates decreased from 8.56 to 7.70 per 100,000, and DALYs reduced from 155.64 to 149.35 per 100,000 between 1990 and 2021, indicative of enhanced healthcare management and resource allocation. Nevertheless, prevalence continued to rise, likely due to factors such as an aging population, improved survival rates (resulting in longer disease duration), enhanced detection, and increasing metabolic risks, including obesity, kidney dysfunction, and poor dietary habits. Germany and Singapore serve as pertinent examples (34). A comprehensive European study further identifies metabolic syndrome as a significant upstream contributor to HHD (34). While moderate-SDI regions exhibit uneven yet notable reductions in disease burden, low-SDI regions continue to experience high prevalence rates due to persistent structural deficiencies in healthcare access and infrastructure (33, 35). Evidence from large trials indicates that lifestyle modification plus pharmacotherapy has been shown to be effective in lowering blood pressure in older adults. Diets rich in vegetables, fruits, whole grains, and low-fat dairy—with limited sugar-sweetened beverages and alcohol—are beneficial; aerobic activity, breathing or isometric training, meditation, and low-sodium, high-potassium diets provide additional gains (36, 37). Beyond blood pressure, targeted interventions to mitigate metabolic risks are critical. Obesity management through sustained weight reduction, structured exercise programs, and pharmacological options (GLP-1 receptor agonists) is associated with reduced HHD risk (38). Addressing kidney dysfunction requires early screening for chronic kidney disease, optimizing blood pressure and glucose control, and wider use of renin-angiotensin system inhibitors (39). Population-wide strategies, including taxation of sugar-sweetened beverages, front-of-pack food labeling, and policies promoting healthier urban environments, can help reduce obesity and related metabolic risks at scale. Health education and self-management remain central delivery vehicles for these strategies, enabling individuals to sustain long-term adherence. Globally, crude prevalence rose from 202.28 to 208.42 per 100,000 between 1990 and 2021. Although mortality and DALYs declined, the reductions were modest—especially in low-SDI regions where resource constraints and weak governance impede control (40). Accordingly, HHD is increasingly contained in many settings, yet low-SDI regions still face substantial challenges.

Trends also vary by geography. In East Asia, crude prevalence increased (EAPC of 1.57), and total cases nearly doubled from 1990 to 2021; however, age-standardized prevalence decreased (EAPC of −0.63), indicating that population growth and aging—rather than higher age-specific risk—drove the absolute increase in cases. In Sub-Saharan Africa, particularly East, Central, and West Africa, age-standardized prevalence remains among the world's highest (291.80–266.25 per 100,000) with EAPCs near zero, suggesting that despite some system improvements, the burden has not been meaningfully alleviated. The persistently high burden is likely influenced by a confluence of structural, socioeconomic, and cultural factors (41). Health-system constraints include shortages of facilities and trained personnel, unreliable supplies of essential medicines and diagnostics, and high out-of-pocket costs that limit access to care (42). It is common for care to be provided by low-skilled health workers, particularly in rural areas, where access to medical services often requires high out-of-pocket payments (43). Rural and peri-urban populations face additional barriers due to long travel distances, poor transportation infrastructure, and shortages of health facilities (44). In urban settings, overcrowded public health clinics frequently result in long waiting times and brief consultations, further discouraging patients from seeking treatment unless symptoms become severe (44). Moreover, health institutions across much of Africa often struggle with limited, inconsistent, or insufficient supplies of anti-hypertensive medications (45). Socioeconomic barriers, such as poverty, food insecurity, and low educational attainment—particularly among women—further reduce health-seeking behavior and adherence to treatment (46). In many cases, hypertension remains undetected until the onset of severe complications such as heart failure, stroke, or kidney disease, reflecting delayed awareness and late-stage diagnosis (47). Cultural influences also play a role, with stigma surrounding cardiovascular conditions, distrust of formal healthcare systems, and reliance on traditional medicine contributing to delays in diagnosis and treatment (48). Insufficient awareness of medical care contributes to reduced treatment adherence (40, 49). Taken together, these barriers help explain why Sub-Saharan Africa still faces high prevalence and relatively limited improvements, despite overall global progress in HHD control.

Notably, the significant gender disparities in HHD burden emerged when examining absolute vs. age-adjusted metrics. While women bore a higher total global case count (2021:6.803 million vs. men's 5.702 million), this largely reflects demographic structure rather than biological risk—a distinction clarified by near-identical age-standardized prevalence (men: 148.86; women: 146.65 per 100,000). The contrast highlights how unadjusted totals can obscure true sex-specific disease patterns. In South Asia, women experienced disproportionately high age-standardized prevalence (141.39 vs. 77.08 per 100,000 in men; Supplementary Table S1). This disparity is driven by several factors. Pregnancy-related conditions, such as hypertensive disorders of pregnancy (including pre-eclampsia), may lead to lasting cardiovascular effects (50). Sociocultural and economic barriers, including restrictive gender norms that limit women's autonomy to seek care and economic dependence that reduces access to timely treatment, further compound risk (51). Unequal healthcare access also plays a role, with underfunded maternal health services, geographic barriers in rural areas, and preferential allocation of household health resources to male family members leading to under-diagnosis and inadequate management in women (52). Together, these challenges illustrate how gender inequities, when combined with weak health systems, may amplify biological risks and contribute to distinct epidemiological profiles. To address these gaps, gender-sensitive interventions are urgently needed, including: strengthened antenatal and postpartum cardiovascular follow-up, programs to increase women's health decision-making autonomy, and policies ensuring equitable access to diagnostics and treatment for women.

Similar to certain healthcare expenditure studies, frontier analysis enables policymakers to understand the efficiency and effectiveness of public health policies, thereby enhancing policy design and implementation (53). Frontier analysis reveals a non-linear relationship between SDI progression and HHD burden. While high-SDI regions generally demonstrate effective burden control—with declining mortality and DALYs indicating robust healthcare capacity—their rising prevalence presents a distinct epidemiological paradox. Several high-SDI countries (Germany, Singapore, and the United States) exhibit increasing burden components despite socioeconomic advantages. Therefore, for populations in high SDI countries, intensified awareness campaigns on HHD risk factors and lifestyle interventions targeting rising obesity rates, poor dietary patterns, and sedentary behavior should be implemented(54, 55). Furthermore, the burden of HHD remains heavy in low-SDI countries and regions, particularly in areas such as Africa, South Asia, and Southeast Asia. Although some low-SDI countries have shown some improvement in controlling the disease, the scarcity of medical resources and the weakness of public health systems present greater challenges for these countries (56). Projections indicate a substantial rise in the global burden of HHD among older adults over the next three decades, driven by population aging. By 2050, age-standardized rates are predicted to reach 251.56 (prevalence), 16.51 (mortality), and 322.04 (DALYs) per 100,000 in the older adults (65+), highlighting the potential threat HHD poses to healthy aging. These trends point to two important policy considerations. First, there is a strong need for global cardiovascular prevention strategies targeting aging populations, particularly to mitigate the “silver tsunami” of age-driven cases. Secondly, While SDI elevation correlates with reduced burden, low-SDI regions face disproportionately severe impacts. Though health inequalities are narrowing globally, significant disparities persist—especially in resource-limited settings where aging compounds existing vulnerabilities. In addition, this study provides novel insights through its application of frontier analysis to quantify HHD inequalities across 204 countries from 1990–2021. Critically, this approach identifies nations under-performing relative to their SDI peers such as high-SDI countries with unexpectedly rising burden, revealing actionable gaps in healthcare delivery. By mapping these frontier deviations, our analysis: delivers evidence-based opportunities for targeted interventions in underachieving regions, highlights how optimal resource reallocation could bridge performance gaps, generates policy-relevant benchmarks for accelerating progress toward health equity. Countries should enhance collaboration to tackle global aging challenges by sharing resources and experiences, researching treatments for HHD, and developing life-cycle management models. Future studies should explore using technologies like AI, telemedicine, and electronic health records to prevent and manage HHD.

The study also employs the advanced GBD 2021 tool, which incorporates a large sample size and complex statistical methods. These limitations should be carefully considered when interpreting our findings. Our study has several limitations that highlight opportunities for enhanced data integration: Data gaps from underreporting and hospitalization bias: GBD 2021 data may underestimate true disease burden, particularly in underdeveloped regions where inadequate hypertension registries, healthcare shortages, and missed diagnoses lead to systematic underreporting (57). Further, its heavy reliance on hospitalized patient data omits milder outpatient cases and undiagnosed populations. These gaps underscore the critical need for integrating community-based surveillance, digital health records, and primary care datasets to capture the full disease spectrum (58, 59). While GBD employs advanced modeling to mitigate data limitations, inherent biases persist due to uneven data quality across countries (diagnostic misclassification, reporting delays, or variable measurement standards) (60). This heterogeneity challenges direct international comparisons. Standardizing data collection protocols and expanding real-world evidence partnerships would strengthen model validity and comparability. Our decomposition of HHD drivers considered only three factors, excluding potentially significant contributors like insurance coverage and education levels. Additionally, the analysis could not account for complex interactions (bidirectional effects) between socioeconomic, clinical, and environmental drivers. Future frameworks should incorporate broader determinants and interaction analyses to better guide precision interventions. The GBD collaboration's rigorous data processing and modeling methods partially offset these constraints. Nevertheless, strategic investments in integrated data ecosystems—spanning community health systems, standardized registries, and multidimensional covariates—are essential to advance global cardiovascular burden assessments.

## 5 Conclusion

The global burden of HHD is rising, driven largely by population aging and shaped by pronounced disparities across age, gender, and geography. High-SDI regions benefit from stronger healthcare systems yet still face increasing prevalence, while low-SDI regions remain constrained by limited resources and weak infrastructure. Addressing these challenges requires coordinated action: policymakers must prioritize equitable resource allocation and embed HHD prevention into healthy aging strategies; clinicians should emphasize early detection and management of hypertension using gender-sensitive and age-appropriate approaches; and public health systems need to expand community programs that promote healthier diets, regular physical activity, and blood pressure control. Strengthening surveillance and data collection in vulnerable settings will further enable targeted interventions. Ultimately, integrated strategies that align robust healthcare delivery with preventive lifestyle measures are essential to reduce the global HHD burden and advance equity in cardiovascular health.

## Data Availability

The original contributions presented in the study are included in the article/Supplementary material, further inquiries can be directed to the corresponding authors.
